# Monolithic fabrication of 1S1R cross-point device using single Ge_x_Te_1-x_ material by controlling composition between memory and threshold switching

**DOI:** 10.1186/s42649-026-00145-7

**Published:** 2026-10-14

**Authors:** Young-Min Kim, Sang-Heon Park, Jihye Lee, Sangyeop Kim, Siwon Park, Jong-Souk Yeo

**Affiliations:** 1 School of Integrated Technology, College of Computing, Yonsei University, 85, Songdogwahak-Ro, Incheon, Yeonsu-Gu 21983 Republic of Korea; 2 BK21 Graduate Program in Intelligent Semiconductor Technology, Yonsei University, Seoul, 03772 Republic of Korea

## Abstract

Three-dimensional (3D) integrated one-selector one-resistor (1S1R) cross-point arrays are emerging as a leading next-generation memory technology. Achieving both nonvolatile memory switching and volatile threshold switching within a single material system offers tremendous potential for simplifying fabrication. This study demonstrates that the memory and threshold switching behavior of germanium-telluride (Ge_x_Te_1-x_) devices can be precisely tuned by controlling their atomic ratio. In the Te-deficient Ge_x_Te_1-x_ system, the device exhibits nonvolatile memory switching with fast operating speed. Conversely, the Te-rich system functions as an excellent bidirectional selector for high-density memory applications, exhibiting high selectivity (> 10^4^) and a short delay time (< 55 ns). Material analyses using X-ray photoelectron spectroscopy and Raman spectroscopy reveal that these distinct electrical behaviors are driven by composition-dependent changes in the atomic structure. Furthermore, the fully integrated 1S1R stack demonstrates robust SET/RESET switching characteristics, maintaining a stable memory window of 0.2 V and an on/off current ratio exceeding two orders of magnitude (~ 1.12 × 10^2^). Notably, this integrated configuration provides superior operational reliability and enhanced endurance (~ 8 × 10^6^ cycles) compared to standalone phase-change memory devices. By elucidating these underlying mechanisms, this work reduces the fabrication complexity of stackable devices and opens up wide-ranging applications for unified memory and selector systems.

## Introduction

Data in the twenty-first century has become an extremely valuable resource. According to the latest study, one person is generating 1.7 MB of data every second in these days (equivalent to global annual data of 429 ZB per year) (Berisha et al. 2022), suggesting that there is still plenty of room for improvements in data storage. Also, in the age of artificial intelligence (AI), such big data is reprocessed through machine learning to generate new value-added data. To generate value from a large collection of data, current mainstream classic memory devices are charge-based memories (Van Houdt 2011), such as dynamic random-access memory (DRAM) (Kim et al., 2018) as a working memory and FLASH memory (Bez et al. 2003) as a storage memory. However, despite the continuous development of DRAM and FLASH memory, there is a limit to physical scaling and there are difficulties in process technology (Mutlu 2013).

To address these limitations, storage-class memory (SCM), a term used by IBM in 2006, which has been proposed to fulfill the performance gap between DRAM and FLASH memory (Burr et al. 2008). SCM provides a main working memory solution, which has similar capacity to storage media. In addition, the ability to read and write data so quickly in SCM enables mixed transaction/analysis and read/write workloads that are necessary for big data analysis and AI applications. Notably, these next generation non-volatile memory devices are mainly two-terminal devices based on resistance changes, unlike the conventional charge-based classic memory (Burr et al. 2010; Wong et al. 2012). Typical examples are resistive switching RAM (ReRAM) (Akinaga et al., 2010), phase change RAM (PCRAM) (Raoux et al. 2008), and magnetic RAM (MRAM) (Dieny et al., 2017).

To effectively complement the existing memory hierarchy, achieving high density and low cost remains the primary challenge. In this situation, three-dimensional (3D) cross-point array architecture has emerged as a breakthrough. Phase change memory (PCM) is widely considered a promising candidate for 3D-stacked architecture due to its excellent cycling endurance, good scalability, and bit-alterable capability (Burr et al. 2016). These two-terminal memory devices use a cross-point architecture and need an additional non-linear conducting selector device in series to eliminate the conducting sneak paths that occur between non-selected cells (Shi et al. 2020).

The cross-point stacked solution combining a PCM with an Ovonic threshold switch (OTS) as a selector was proposed in 2009 (Kau et al. 2009). In 2015, Intel and Micron announced the production of a new memory that is ten times denser than conventional DRAM and 10^3^ times more durable than FLASH memory (Bairey 2015). Recent studies have further focused on engineering chalcogenide-based OTS materials to improve selector performance and elucidate switching mechanisms. Doping strategies, such as nitrogen incorporation into SiTe-based OTS devices, have been shown to enhance selectivity by suppressing leakage currents (Lee et al. 2024). Furthermore, nanoscale characterization techniques based on atomic force microscopy have provided critical insights into the structural evolution of GeTe-based OTS devices during switching, revealing nanocrystalline formation at the electrode interface after the first-firing operation (Kim et al. 2026). While the one-selector one-resistor (1S1R) device comprising PCM and OTS has demonstrated competitive performance as a top contender in the arena of SCM, its cost structure remains a significant barrier. The primary challenge in reducing the fabrication costs of the integrated 1S1R structure lies in the complexity of material compositions. It commonly requires different conditions optimized for PCM and OTS in the manufacturing processes. In light of this, the monolithic fabrication of integrated architecture using simple material systems is a promising strategy for minimizing manufacturing costs.

In this work, we fabricated a monolithic 1S1R device based on a single Ge_x_-Te_1-x_ binary system. The electrical behavior of the binary system is modified by tuning the chalcogenide composition. Te-rich compositions exhibit OTS characteristics, while near-stoichiometric compositions show non-volatile memory switching. Through the results from the X-ray photoelectron spectroscopy (XPS) and Raman spectroscopy, we confirm that the functional transition from PCM to OTS behavior is driven by the microscopic change in local bonding networks into the Te–Te rich matrix in the Ge_x_-Te_1-x_ binary system. The resulting monolithic 1S1R devices show excellent operational stability with endurance of ~ 8 × 10^6^ cycles and voltage margin of ~ 0.2 V under operating conditions of less than 1 V.

## Materials and methods

Ge_x_Te_1-x_ thin films were deposited using an RF magnetron co-sputtering method with Ge (99.999%) and Te (99.999%) single targets onto a SiO_2_/Si substrate for evaluating its chemical composition and crystallinity. Prior to deposition, the main chamber was evacuated to a base pressure below 1.0 × 10^–7^ Torr. Sputtering was performed in a high-purity argon (Ar) atmosphere at a working pressure of 1.0 × 10^–2^ Torr. The film composition was controlled by adjusting the RF power applied to each target. The sputtering power used for the Te target was 15 W, while that used for the Ge target varied from 20 to 50 W in 5 W increments to achieve the desired Ge_x_Te_1-x_ (Ge_0.11_Te_0.85_ to Ge_0.77_Te_0.23_) films. The chemical composition was evaluated by XPS, Raman spectroscopy, and in-situ X-ray diffraction (in-situ XRD). The device configuration was confirmed using transmission electron microscopy (TEM) (JEM-ARM 200 F NEOARM (JEOL Ltd., Japan)).

PCM and OTS devices with hole-structures were fabricated to investigate electrical characteristics. First, a tungsten (W) bottom electrode (BE) with 100-nm thickness and areas from 34 nm × 34 nm to 1921 nm × 1921 nm was used. Then, the Ge_x_Te_1-x_ films with a thickness of 20 nm were deposited employing the RF magnetron co-sputtering method. Subsequently, a W top electrode (TE) with 100-nm thickness was deposited via DC magnetron sputtering. Device patterns were defined using maskless lithography (Nanosystem Solutions, Inc., UK) with AZ-5214E photoresist. The electrical properties were measured through a Keithley 4200 A analyzer (Keithley) at room temperature.

### Single Ge_x_Te_1-x_ material for both memory switching and threshold switching

Recently, the importance of OTS has been increasing as a selector device in high-density memory devices, and it is being used as an essential component in PCM-based memory system. However, research on Ge or Si-based chalcogenide materials used in OTS is relatively lacking compared to PCM materials. Although the two devices are based on the same chalcogenide materials, they exhibit fundamental differences in their operating mechanisms. While PCM implements memory operation by utilizing structural phase transitions between crystalline and amorphous phases (Wuttig et al., 2007), amorphous OTS materials exhibit threshold switching characteristics through electronic transitions without crystallization (Adler et al. 1978).

**Fig. 1:**
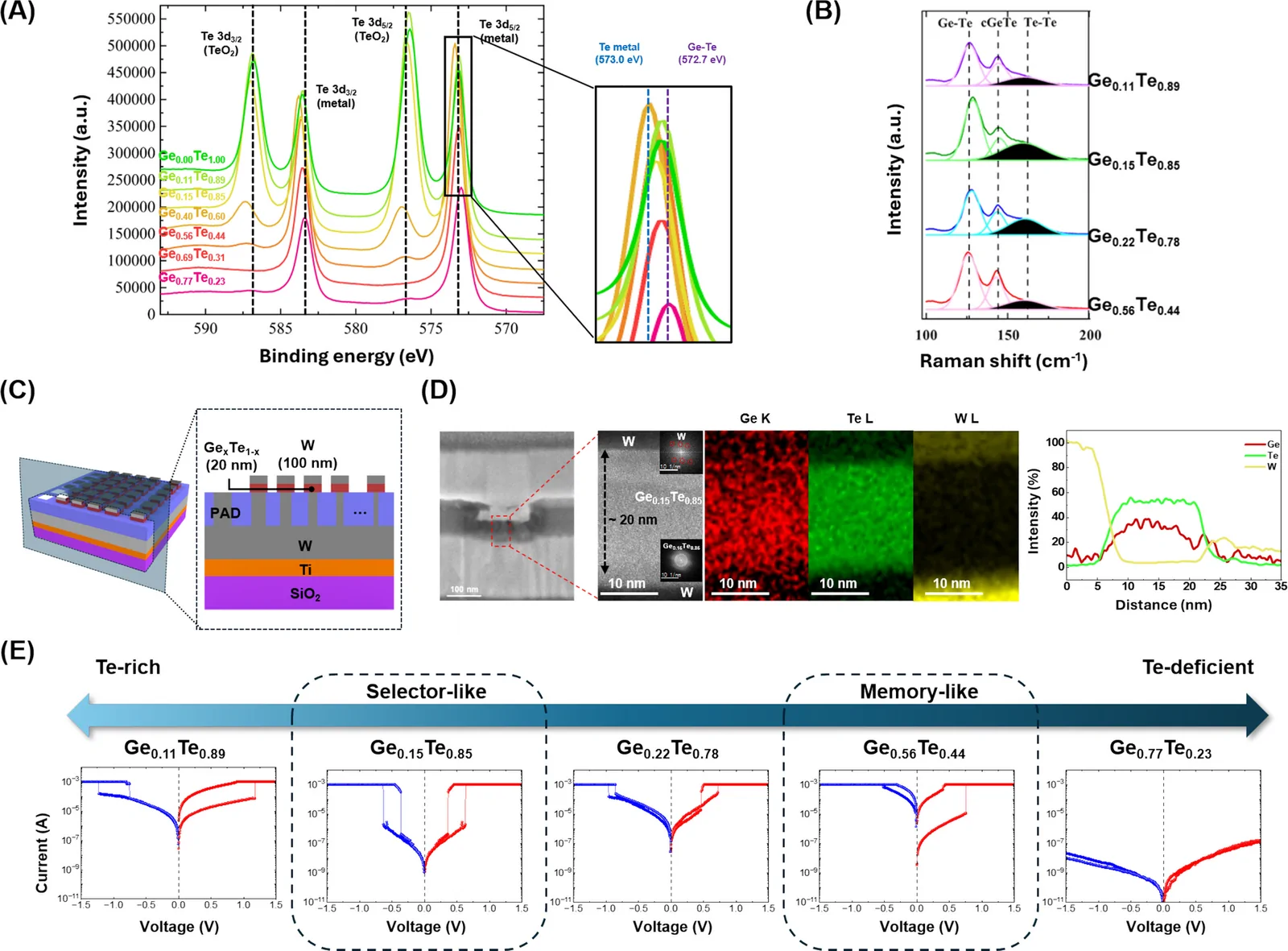
Ge_x_Te_1-x_ thin film characterization and device electrical properties. **A** XPS spectra of Te3d of Ge_x_Te_1-x_ chalcogenide films. **B** Raman spectra of Ge_x_Te_1-x_ chalcogenide films. **C** Schematic illustration of oxide layer hole etched device. **D** TEM and EDS mapping images of the fabricated Ge_0.15_Te_0.85_ device. **E** DC I-V electrical characteristics by controlling the composition of Ge_x_Te_1-x_ films (Ge_0.11_Te_0.89_, Ge_0.15_Te_0.85_, Ge_0.22_Te_0.78_, Ge_0.56_Te_0.44_, and Ge_0.77_Te_0.23_)

Since the amorphous single binary Ge_x_Te_1-x_ systems have various band structures and bonding characteristics significantly different from those of the crystalline structure, it is possible to control memory switching characteristics in PCM and threshold switching characteristics in OTS. Furthermore, these band structure and bonding characteristics depend on the composition of the thin film. To evaluate the thin film characteristics, 100 nm-thick co-sputtered Ge_x_Te_1-x_ films were deposited on Si/SiO_2_ substrates. Figure 1 shows the material properties such as crystallinity and bonding characteristics of the Ge_x_Te_1-x_ thin film of 100 nm, and electrical characteristics of the Ge_x_Te_1-x_ thin film of 20 nm.

XPS spectra of Ge *3d* and Te *3d* peaks are identified according to the XPS handbook (Moulder et al., 1992). Figure 1A shows gradual change of bonding characteristics with increasing Te content. XPS spectra of Te *3d* clearly indicate Ge-Te and Te–Te bonds at approximately 572.7 eV, and Te–O bonds at 576.9 eV (Fig. 1A). These observations of Ge-Te, Te–Te, and Te–O bonds are consistent with previously reported literature (Yashina et al. 2008). This result clearly shows that Ge-Te bonding is energetically more favorable than Te–Te or Te–O bonding in Te-deficient Ge-Te system at least up to Ge_0.56_Te_0.44_. Notably, the Te–O peak remained absent until the Te content reached Ge_0.56_Te_0.44_, however, it appeared at Ge_0.40_Te_0.60_ and its intensity gradually increased with further Te enrichment. This suggests that as the Te concentration increases, the tendency to form Te–Te and Te–O bonds increases. Specifically, the peak at 576.9 eV intensifies and shifts to higher binding energy with increasing Te-atomic percentage. This trend can be attributed to the fact that when the Te concentration is low, most Te atoms are consumed in Ge-Te bonds due to the chemical affinity between Ge and Te (Lee 2025). As the Te content increases beyond the coordinative saturation point of Ge, the excess Te atoms that do not participate in Ge-Te bonding instead facilitate the formation of Te–Te and Te–O bonds. Consequently, while Te primarily participates in heteropolar Ge-Te bonds in the Te-deficient compositions, the excess Te in Te-rich compositions leads to formation of homopolar Te–Te bonds. These Te–Te bonds contribute to an increased density of localized trap states within the mobility gap, which is widely understood threshold switching behavior in the amorphous chalcogenide system (Hatayama et al. 2023; Qua et al., 2024). Furthermore, our previous study demonstrated that the p-lone pairs of Te–Te bonds affect the electronic structure and trap distributions in Te-based OTS devices (Park et al. 2026).

Figure 1B shows the distinct phases present in Ge_x_Te_1-x_ compounds. Raman spectra of amorphous materials are similar to crystalline materials with relatively broad peaks. The broadening in Raman spectra is attributed to inherent disorder within the amorphous alloy. It is evident from the spectra that this structural disorder increases with Te content, as indicated by the broadening of the full-width at half-maximum (FWHM) of the peaks. In our samples, Raman bands were observed at 127.2, 141.8, and 168.0 cm^−1^. It is reported that the amorphous GeTe give peaks at wave numbers of 83, 125, 162, and 218 cm^−1^ from GeTe_4-n_Ge_n_ tetrahedral units (Andrikopoulos et al. 2006) and crystalline GeTe shows dominant peaks at about 80 and 120 cm^−1^ (Qu et al. 2020). From bulk Te spectra, a peak at 121 cm^−1^ representing the A_1_ mode was identified, along with two low-intensity peaks at 140.8 and 92.5 cm^−1^ corresponding to E_TO_ modes in crystalline Te–Te chain (Majumder et al. 2025). The prominent peaks observed in our Te-rich Ge-Te system result from these Te–Te chains and interconnected Ge-Te bonds. To further elucidate the local structure, Raman spectra were deconvoluted into multiple Gaussian peaks corresponding to specific vibrational modes. The positions of peaks in Raman spectrum of crystalline GeTe state can be assigned to tetrahedral, distorted, and defective octahedral Ge sites. On the other hand, peaks in amorphous GeTe state can be attributed to tetrahedral and defective octahedral Ge sites (Upadhyay et al. 2011). Hence, this result supports that amorphous GeTe peak intensity gradually increased with increasing Te content. This indicates that Ge-Te bonding is dominant at low Te concentrations; however, as the proportion of Te increases, the excess Te atoms, which make the film amorphous, can contribute to the formation of a conductive path, perfectly matching prior reports.

In order to fabricate highly reliable cross-point devices, we developed SiO_2_ layer with 18 different sizes of etched holes (ranging from 34 nm × 34 nm to 1921 nm × 1921 nm) with 10-nm-thick adhesion layer (Ti) and passivation layer (SiO_2_). The optimized device structure is illustrated in Fig. 1C. All unit devices were fabricated with an identical structure, and cross-sectional TEM image was employed to confirm the device structure and film thickness using the representative Ge_0.15_Te_0.85_ film condition (Fig. 1D). The hole-etched structure is clearly visible in the cross-sectional TEM image, and the 20-nm-thick Ge_x_Te_1-x_ film acts as a functional layer between the top and bottom electrodes. Energy dispersive X-ray spectroscopy (EDS) was used to analyze the composition of each region. Ge and Te was detected in the EDS spectra in functional layer and there were no remarkable differences among regions.

On the basis of all previous studies of Ge_x_Te_1-x_ material system, we here selected prototypical compositions to evaluate the impact of the compositional distribution on their electrical characteristics: Ge_0.11_Te_0.89_, Ge_0.15_Te_0.85_, Ge_0.22_Te_0.78_, Ge_0.56_Te_0.44_, Ge_0.77_Te_0.23_ (Fig. 1E). Among them, the memory switching and ovonic threshold switching were confirmed in the Ge_0.56_Te_0.44_ (GeTe) and Ge_0.15_Te_0.85_ (GeTe_6_), respectively.

### Phase change memory device based on GeTe

GeTe has been integrated into a simplified device structure that we introduced with a 100-nm-thick W BE in direct contact with a 20-nm-thick GeTe layer. The cell stack was completed with a 100-nm-thick W TE layer. To initialize the device in the SET state prior to electrical characterization, a post-thermal annealing process was performed at 230℃ in an N_2_ atmosphere.

**Fig. 2:**
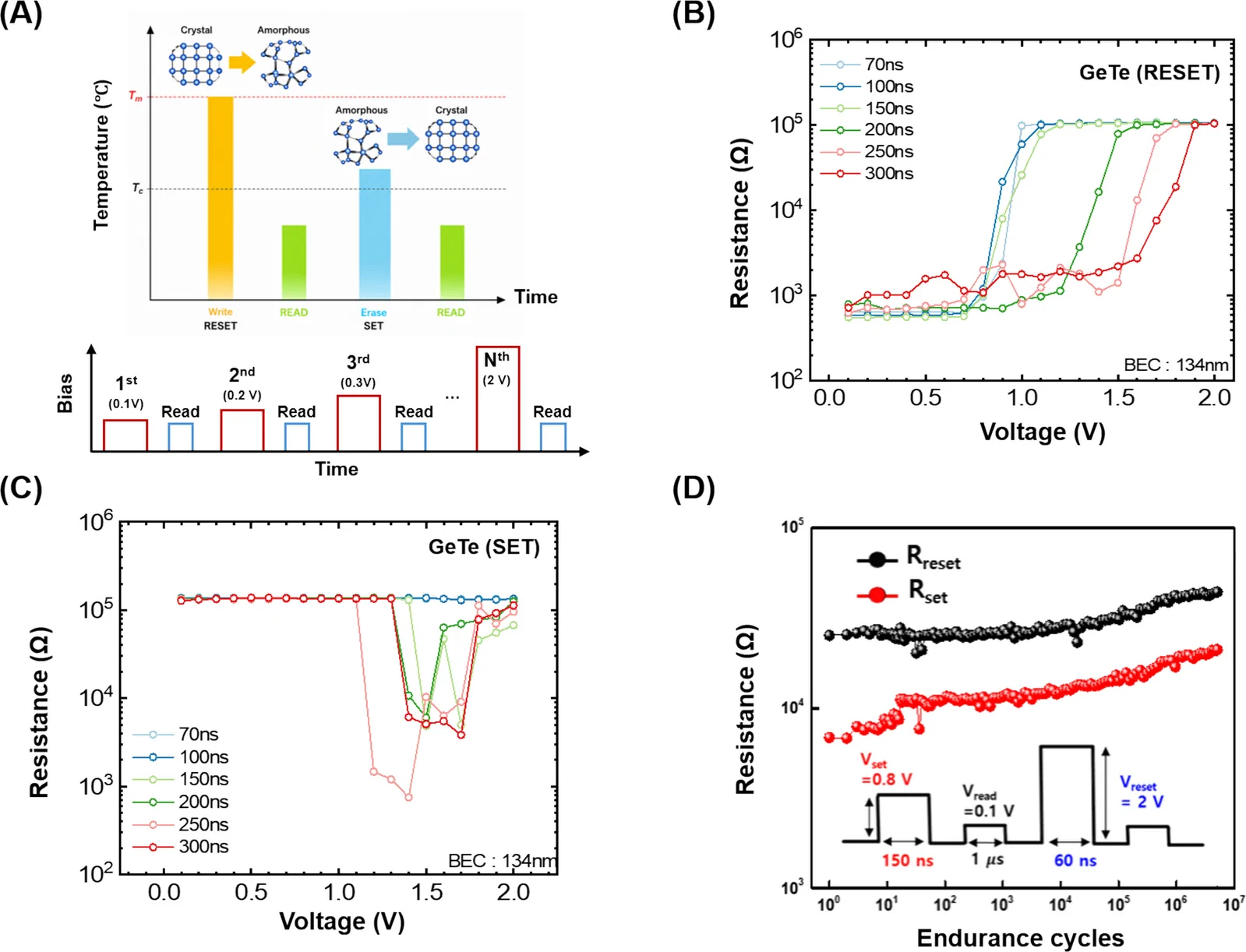
**A** Schematic illustration of PCM working strategy. **B** R-V characteristics in RESET operation of GeTe-based PCM device (BEC: bottom electrode contact). **C** R-V characteristics in SET operation of GeTe-based PCM device.** D** Endurance test of GeTe-based PCM device

Figure 2A illustrates the operational strategy of the PCM. Data storage in PCMs relies on the electrical resistance contrast between the crystalline and amorphous phases. The RESET (writing a ‘0’) state corresponds to the crystalline-to-amorphous transition, while the SET (writing a ‘1’) state utilizes the amorphous-to-crystalline transition. Since amorphization is a faster process than crystallization, the RESET operation requires a high temperature of short duration for melting the material into an amorphous phase, then this state is quenched quickly to freeze the atoms in a disordered structure. In contrast, the SET process requires longer pulse duration to provide sufficient time for the atoms to rearrange into a stable crystalline structure.

The programming characteristics were evaluated using PCM devices with a 134-nm BE contact size through a pulse generator and an active probe. The cell current was measured on a 100-Ω load resistor for pulses down to 50 ns (with 10 ns rise/fall times). We applied voltage pulses ranging from 0.1 V to 2.0 V in 0.1 V increments, accompanied by a read voltage pulse (Fig. 2A). Figure 2B and C show the R-V characteristics of the PCM during the RESET and the SET operations, respectively. The pulse duration for RESET and SET varied from 70 to 300 ns. Figure 2B shows that GeTe exhibits reliable amorphization over the entire pulse-width range, maintaining a minimum resistance contrast of two orders of magnitude between the RESET and the SET states. However, the voltage required for RESET operation increases with increasing pulse width. Considering power-consumption efficiency, pulse widths of less than 70 ns are preferable because stable RESET switching can be achieved at relatively low voltages. Figure 2C shows that the SET operation exhibits stable crystallization behavior at pulse widths of 150 ns and longer, yielding a resistance change exceeding two orders of magnitude. Therefore, a pulse width of 150 ns is appropriate to perform the SET operation with minimum programming time. Endurance of the device was evaluated using 60-ns and 150-ns pulse widths for RESET and SET operations, respectively. Figure 2D demonstrates that the GeTe-based PCM device maintains a stable resistance contrast of approximately one order of magnitude between the RESET and SET states without device failure up to ~ 5 × 10^6^ cycles.

### Ovonic threshold switching device based on GeTe_6_

GeTe_6_ was integrated into a simplified device architecture, as previously described, consisting of a 100-nm-thick W BE, a 20-nm-thick GeTe_6_ layer, and a 100-nm-thick W TE.

**Fig. 3:**
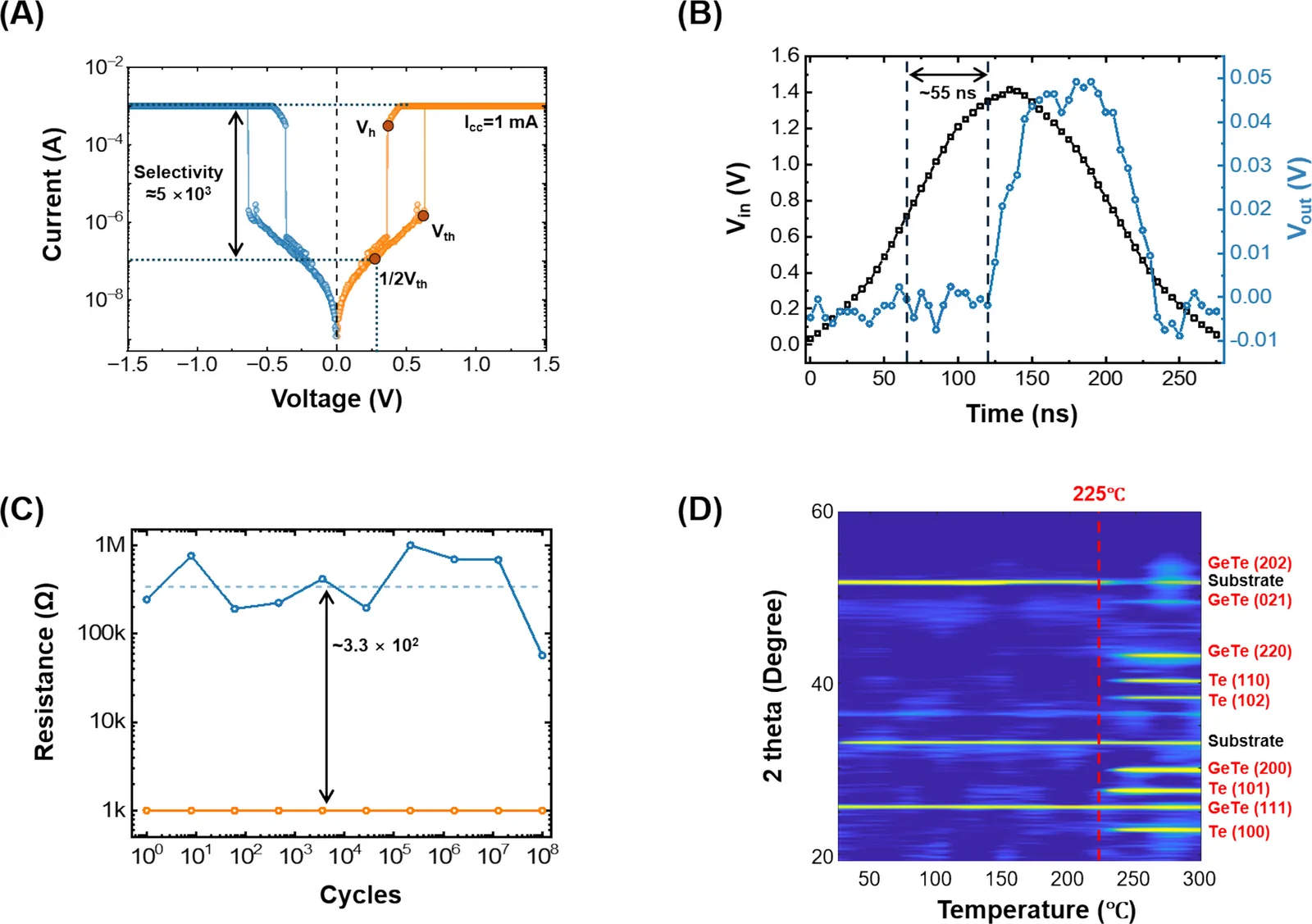
**A** I-V characteristics of GeTe_6_-based OTS device (Compliance current, I_cc_ = 1 mA). **B** Delay time of threshold switching in GeTe_6_-based OTS device. **C** Endurance test of GeTe_6_-based OTS device.** D** In-situ XRD analysis of GeTe_6_ film

Figure 3A shows the I-V curves of the GeTe_6_-based OTS device, demonstrating excellent bidirectional selector characteristics with high resistance at low voltage and low resistance at high voltage. The device exhibits field-dependent resistance changes in both bias polarities. The resistance change occurring at specific threshold voltage (*V*_th_) is instant, volatile, and abrupt, allowing the state of the OTS device to be precisely controlled by applied electric field on the device. When applied voltage (*V*_a_) is small (*V*_a_ < *V*_th_), the GeTe_6_-based device is in high-resistance state (HRS), referred to as the OFF state. This state (higher than 1 GΩ) effectively suppresses leakage currents. When the voltage is high (*V*_a_ > *V*_th_), an OFF-to-ON transition is triggered, causing the resistance drop by several orders of magnitude as the device becomes low-resistance state (LRS). The low resistance of the fabricated GeTe_6_-based OTS device in LRS (lower than 1 kΩ at 1.0 V) is able to supply currents up to several milliamps for programming/erasing operation to an adjacent memory device. Due to the volatile nature as mentioned above, it is maintained only as long as the *V*_a_ exceeds a specific voltage, referred to as holding voltage (*V*_h_). When *V*_a_ becomes lower than specific *V*_h_, an ON-to-OFF transition occurs, and the device recovers its initial HRS. These electrical characteristics are suitable for enhancing reliability in high-density memory arrays because the OTS device can provide high operation current to selected memory cells while inhibiting sneak currents to unselected memory cells.

The GeTe_6_-based OTS device exhibits a sharp switching slope, which is desirable for a large operation window and low power consumption in memory array. Figure 3B shows the rapid response speed of the device to external voltage (< 55 ns of delay time). The reliability of the device under electrical or thermal stress was systematically evaluated. The endurance test demonstrates excellent electrical stability, demonstrating stable operation without breakdown for 10^8^ cycles using 150-ns pulses (Fig. 3C). In addition, in-situ XRD analysis confirmed high thermal stability up to 225 ℃ (Fig. 3D). After annealing at 225 ℃, the GeTe_6_ film became crystalline, resulting in the loss of its OTS electrical characteristics.

### 3D cross-point 1S1R device based on stacked GeTe/GeTe_6_

**Fig. 4:**
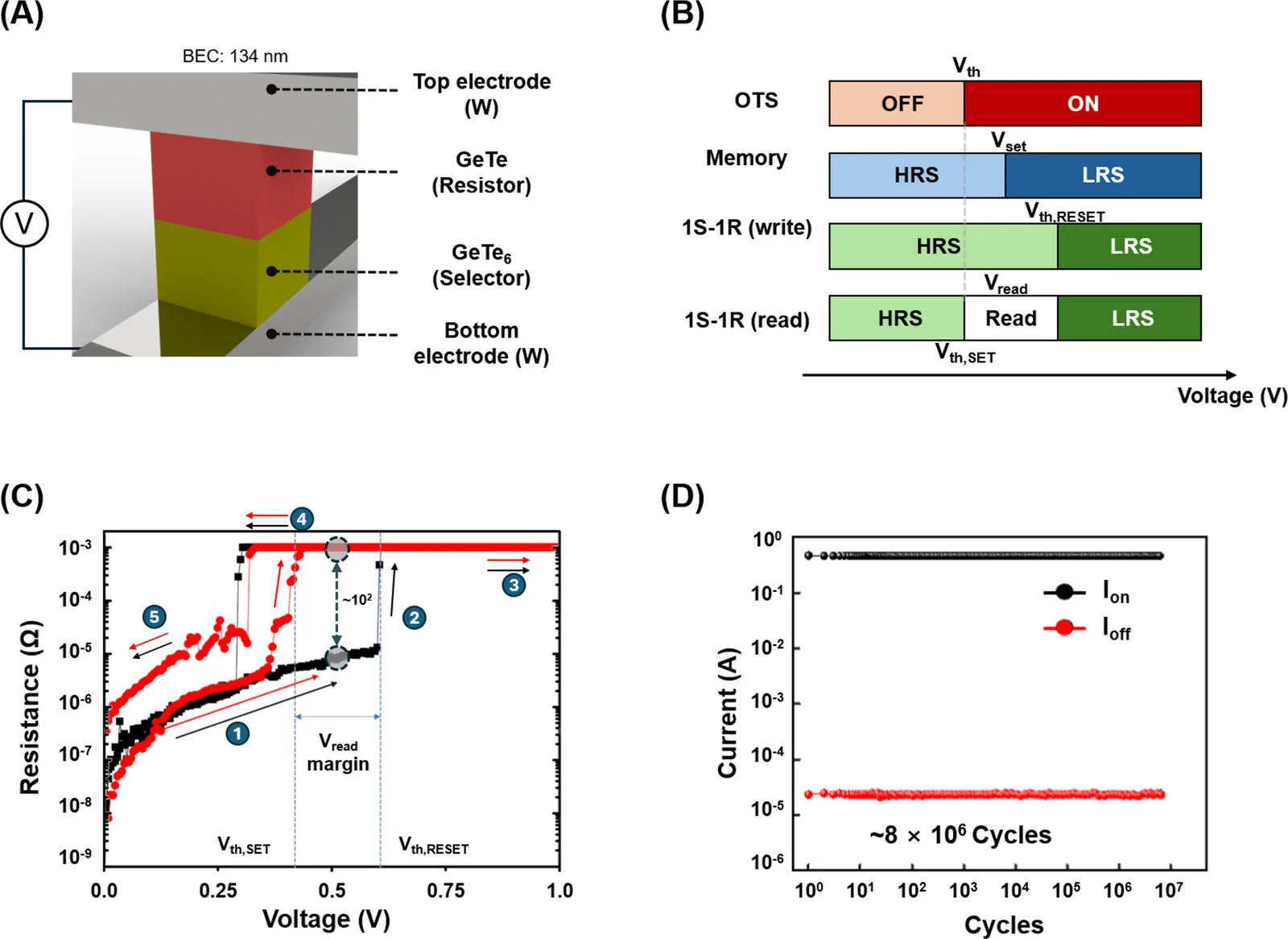
**A** Schematic illustration of GeTe/GeTe_6_ stacked 3D 1S1R cross-point device. **B** OTS/PCM stacked 1S1R cross-point device working strategy **C** I-V characteristics of GeTe/GeTe_6_ stacked 1S1R cross-point device (Compliance current, I_cc_ = 1 mA). **D** Endurance test of GeTe/GeTe_6_ stacked 1S1R cross-point device

On the basis of understanding GeTe-based PCM device and GeTe_6_-based OTS device, we fabricated a monolithic 1S1R stacked device based on GeTe/GeTe_6_ films for neuromorphic applications (Fig. 4A). When PCM is in the SET state, the specific voltage (*V*_th,SET_) at which a sharp current increase occurs in 1S1R device is nearly identical to *V*_th_ of the OTS-only device. This is because the PCM with low resistance in the SET state acts as a negligible voltage divider. As a result, most of the applied voltage drops across the OTS layer, making *V*_th,SET_ approximately equal to *V*_th_ of a device with only OTS. In contrast, when the PCM is in the RESET state, the specific voltage (*V*_th,RESET_) at which a sharp current change occurs is higher than *V*_th,SET_ because both the selector and high-resistance PCM must be turned ON simultaneously. Based on these electrical properties, the operational strategy of PCM/OTS stacked 1S1R cross-point device is illustrated in Fig. 4B. Figure 4C presents the I-V characteristic of the 1S1R device operated using the described strategy. As expected from the operating principle, the *V*_th,RESET_ required to turn ON the 1S1R device is higher than the *V*_th,SET_. A read voltage (*V*_read_) can be chosen between ~ 0.4 V (*V*_th,SET_) and ~ 0.6 V (*V*_th,RESET_). This yields a current ratio exceeding two orders of magnitude (~ 1.12 × 10^2^), enabling reliable state discrimination between the SET and RESET states. This device shows the memory window of ~ 0.2 V and the great reliability with high uniformity over the ~ 8 × 10^6^ cycles (Fig. 4D). Interestingly, the integrated 1S1R device exhibited enhanced endurance characteristics compared to the standalone PCM device, which has also been reported in the other study (Yeh et al. 2018). This improvement can be attributed to the intrinsic current-limiting effect (self-compliance) of the GeTe_6_-based OTS layer, which effectively prevents excessive Joule heating and subsequent material degradation in the PCM layer (Wang et al. 2021). The monolithic chalcogenide-on-chalcogenide interface also contributes to improved thermal confinement and reduced atomic diffusion compared to conventional metal-electrode contacts, resulting in superior operational reliability (Yang et al. 2021). On the other hands, chalcogenide-based OTS devices are known to suffer from atomic diffusion or phase segregation as a primary degradation mechanism during repeated switching operations (Wang et al. 2022; Park et al. 2025). Although direct microscopic observation of the GeTe/GeTe_6_ interface is not currently included, the possibility of interdiffusion between the two functional layers under repetitive electrical stress cannot be entirely ruled out. Therefore, establishing a stable interfacial barrier between the OTS and PCM layers may represent a promising strategy for maintaining integrated device performance (Chien et al. 2019).

## Conclusions

In summary, this study demonstrates the monolithic integration of a 3D 1S1R cross-point architecture using a single chalcogenide binary Ge_x_Te_1-x_ system. Through precise control of the material compositions using RF magnetron co-sputtering, we successfully fabricated a GeTe PCM device and a GeTe_6_ OTS selector. Structural and chemical analysis through XPS and Raman spectroscopy confirmed that the functional transition between PCM and OTS is driven by the modulation of local bonding states, shifting from Ge-Te dominant to Te–Te bonding-rich configurations. The integrated 1S1R stack exhibited robust SET and RESET threshold switching with a stable memory window of 0.2 V and a sensing current margin exceeding two orders of magnitude (~ 1.12 × 10^2^). Crucially, the 1S1R configuration demonstrated superior operational reliability and enhanced endurance (~ 8 × 10^6^ cycles) compared to the standalone PCM device. This improvement was attributed to the intrinsic self-compliance effect of the integrated OTS layer, which protects the memory element from electrical and thermal overstress during repetitive cycling. In addition, the 1S1R cross-point device was fabricated in a monolithic lithographic step combining the PCM and OTS films optimized within a single device. This monolithic approach can significantly reduce the fabrication complexity in stackable devices while enabling a low-cost device structure by eliminating separate lithography steps and performing deposition processes in a single cluster system. Our work provides insights into the switching mechanisms of the Ge_x_Te_1-x_ system and offers a scalable, energy-efficient solution for next-generation memory and neuromorphic computing applications.

## Data Availability

The datasets used and/or analyzed during the current study are available from the corresponding author on reasonable request.
